# Changes in Consumption Expenditures among Baby-boomers and Young-olds: Latent Transition Analysis

**DOI:** 10.1093/geroni/igab046.3284

**Published:** 2021-12-17

**Authors:** Si Young Song, Hey Jung Jun, Susanna Joo, Do Kyung Yoon

**Affiliations:** 1 Yonsei University, Seoul, Seoul-t'ukpyolsi, Republic of Korea; 2 Yonsei University, Seodaemun-gu, Seoul-t'ukpyolsi, Republic of Korea

## Abstract

The aim of this study was to examine the longitudinal transition of consumption expenditures among both baby-boomers and young-olds in South Korea. We used data from the 6th (2016) and the 7th (2018) waves of the Korean Longitudinal Study of Ageing (KLoSA). The final sample comprised 1,806 baby-boomers (age range=53-61 in 2016) and 1,483 young-olds (age range=65-74 in 2016). Consumption expenditures were observed with nine types of expenses: food, eating out, public education, private education, housing, health-care, clothing, cultural entertainment, and savings. According to the results from latent transition analysis (LTA), three consumption subgroups were identified among baby-boomers: “non-expenditure for education (NE, 69.7%)” group, “high-public education expenditures (PE, 10.7%)” group, and “high-public and private education expenditures (PPE, 19.6%)” group. For baby-boomers, NE and PE were more likely to remain the same type throughout the two waves, and PPE was most likely to move to NE two years later. Meanwhile, the consumption expenditures of young-olds were divided into “low-saving (LS, 63.7%)” group, “high-saving (HS, 40%)” group, and “education cost-centered (EC, 5.3%)” group. In the case of young-olds, the transition between the groups was unlikely to occur across the two waves which can be interpreted as having fewer life cycle changes than baby-boomers. This study suggests that it is necessary to take into account the difference between the generations when understanding longitudinal transition of consumption expenditures.

